# Heart Rate Variability after Off-Pump versus On-Pump Coronary Artery Bypass Graft Surgery

**DOI:** 10.4061/2009/295376

**Published:** 2009-09-01

**Authors:** Nenad Lakusic, Valentina Slivnjak, Franjo Baborski, Dusko Cerovec

**Affiliations:** Department of Cardiology, Hospital for Medical Rehabilitation, Gajeva 2, HR - 49217 Krapinske Toplice, Croatia, Croatia

## Abstract

*Background.* It is known that after coronary artery bypass graft surgery (CABG) heart rate variability (HRV) becomes significantly decreased with a gradual recovery in a few months after surgery. However, literature data about the impact of the off-pump CABG on postoperative HRV are not complete. Therefore, the aim of this study was to analyze postoperative value of HRV in CABG patients operated on with off-pump versus on-pump coronary surgery. *Methods.* This study included 206 consecutive patients who underwent CABG. Sixty six patients (32%) were operated on off-pump while 140 patients (68%) were operated on using the machine for extracorporal circulation. HRV was analyzed from 24-hours Holter electrocardiogram recordings. *Results.* No significant differences in postoperative values of HRV variables were found between off-pump versus on-pump CABG patients (Mean RR interval 885 ± 106 versus 879 ± 125 ms, standard deviation of all normal R-R intervals 107 ± 30 versus 105 ± 34 ms, NS, total power 2298 ± 2472 versus 2156 ± 1913 ms^2^, NS). *Conclusions.* The results of the study showed that there are no differences in HRV few months after surgery between patients operated on with off-pump versus on-pump CABG.

## 1. Introduction

Today fewer patients undergo coronary artery bypass grafting (CABG) because of modern way of treating coronary artery disease, especially with percutaneous coronary intervention (PCI) procedures. CABG is reserved for patients with significant stenosis of left main, for those with diffuse coronary artery disease, especially for diabetic patients in which “in stent” restenosis is very high and for patients with stenosis at arteries branching [1]. In the last years, CABG was often performed on a beating heart, without machine for extracorporal circulation. The off-pump operation is equally safe as the on-pump CABG [2]. There are reports on some advantages of the off-pump operation compared to the conventional on-pump cardiac surgery [3, 4]. 

 It is known that after cardiac surgery procedures heart rate variability (HRV) becomes significantly decreased [5–7] with a gradual recovery in a few months after surgery [8, 9]. However, literature data about the impact of the off-pump CABG on postoperative HRV are not complete [10]. Therefore, the aim of this study was to analyze postoperative value of HRV in CABG patients operated on with off-pump versus on-pump coronary surgery.

## 2. Material and Methods

This nonrandomized study included 206 consecutive patients who underwent CABG. The study was conducted from December 2005 to February 2007 during the second phase of stationary cardiac rehabilitation. Hospital Ethics committee approved the protocol of the study. The patients were acquainted with the protocol of the study, and written informed consent was obtained from each patient. 

 Inclusion criteria were: CABG patients under 75-years old and stable sinus rhythm. 

 Exclusion criteria were patient older than 75, persistent or permanent atrial fibrillation, frequent ventricular ectopic activity, sinus sick syndrome or atrioventricular block second or third degree, cardiac pacing, CABG at the same time with an implanted artificial valve, acute heart failure or other acute disease which requires interruption of rehabilitation, chronic disease which can have an impact on HRV variables such as chronic renal insufficiency and so forth. 

 All involved patients underwent 3 weeks of stationary cardiac rehabilitation (average 19 ± 2 days). The rehabilitation program consisted of regularly conditioning on the ergocycle, group exercises under a supervision of physiotherapist, individual walks, diet, sessions of psychotherapy, correction of risk profile and optimization of medications [11]. 

 During rehabilitation, 24 hours Holter electrocardiogram (ECG) was performed on all patients, and HRV was analyzed from its recordings. The time from the operation to recording Holter ECG and measuring HRV was 3.7 ± 1.3 months (off-pump versus on-pump CABG patients, *P* = .54). All HRV variables were measured through the 23.2-hour period (ranged 21–24 hours). Ambulatory ECG recordings were made by 3-channel Medilog Digital Holter recorders FD3, Oxford, with 1024 Hz resolution. HRV was analyzed by computer and over-read manually. A commercial system (Oxford Instruments, with software Excel ECG Replay System—Rel. 8.5) was used. Algorithms for arrhythmia analysis gave a label to each QRS complex. An operator cleaned all recordings from artifacts, reviewed beats and modified them if needed, under the cardiologist supervision. Only recordings with less than 15% of ectopic beats were used. Periods with the highest and lowest average R-R intervals, detected from R-R interval histograms, were always validated. The corrected data were processed and HRV was computed. Raw tachogram was used for time domain analysis. The power spectral analysis was computed using fast Fourier transformation. R-R intervals that included ectopic beats were excluded and extrapolated by linear interpolation for the spectral analysis [12–14]. Most of the variables proposed by the Task Force on the HRV were analyzed [15]. Time domain analysis included: Mean RR—mean of R-R intervals for normal beats, SDNN—standard deviation of all normal R-R intervals, SDANN-standard deviation of the 5-minute means of R-R intervals, SDNN-i—mean of the 5-minute standard deviations of RR intervals, rMSSD-square root of the mean of the squared successive differences in R-R intervals and pNN50—percentage of R-R intervals that are at least 50 milliseconds different from the previous interval. Frequency domain analysis covered: TP—Total power (0.0–0.5 Hz), VLF—very low (0.003–0.04 Hz), LF—low (0.04–0.15 Hz) and HF-high (0.15–0.4 Hz) frequency components, with LF/HF—low to high frequency ratio. 

 Apart from the 24-hours Holter ECG, symptom limited exercise test and complete transthoracic echocardiography (Aloka ProSound SSD 5500) was performed during rehabilitation on every patient. 

 In statistical analysis of the obtained results the commercial system SAS System for Windows, Version 6.12 was used. Normality of distribution of the certain variables was tested with the Kolmogorov-Smirnov test. The results have been expressed by mean value ± standard deviation. The chi-square test was used to analyze the differences between certain observed proportions. Differences between groups of patients were tested by the Mann-Whitney test. A *P*- value less than .05 is considered statistically significant.

## 3. Results

The mean age of patients was 61 ± 8 years, ranging from 44 to 74 years. There were 165 males (80%) and 41 females (20%). Sixty six patients (32%) were operated on off-pump and 140 patients (68%) were operated on using the machine for extracorporal circulation. The mean number of bypass performed during the CABG was 2.9 ± 1.1 median 3, ranging from 1 to 6 bypass. Table 1 shows the differences in the primary characteristics of the off-pump versus on-pump CABG patients. 

 During the stationary cardiac rehabilitation, the following values of HRV variables were measured in off-pump versus on-pump CABG patients (Table 2). There were no significant differences in any postoperative value of HRV variables between off-pump versus on-pump CABG patients. 

 The cutoff point for normal overall HRV in general cardiology patient population was 93 milliseconds for SDNN [14]. Twenty-three off-pump (35%), and 51 on-pump CABG patients (36%) had value SDNN <93 milliseconds, (*P* = .75). 

 During the stationary cardiac rehabilitation, the ejection fraction (EF) of the left ventricle in the group of on-pump CABG patients was 59 ± 9% (with a range of 23%–66%), and in the off-pump group of CABG patients was 60 ± 10% (with a range of 26%–70%), (*P* = .82). Mean value of a workload achieved on symptom limited exercise test performed at the end of rehabilitation in on-pump was 113 ± 37 W or 6.3 ± 1.2 metabolic equivalents (METs) versus off-pump group of CABG patients 114 ± 29 W or 5.8 ± 1.1 METs (*P* = .77 for W and *P* = .43 for METs).

## 4. Discussion

The main results of this study show that patients after cardiac coronary surgery depending on using the machine for extracorporal circulation have similar values of HRV. In other words, there were no significant differences in either one of the analyzed variables of HRV between the groups of off-pump versus on-pump patients few months after surgery. It is well known that after CABG HRV becomes significantly decreased [6, 7] with a gradual recovery in a few months after surgery [8, 9]. The possible reasons for decreased HRV after CABG is a combination of surgical manipulation during the operation on the heart and other anatomic structures around the heart, anesthesiology procedures, duration of cardioplegia, and also extracorporal circulation and so forth. [16–18]. 

 Kalisnik et al. [10] found out that after off-pump CABG cardiac autonomic function is better preserved 7 days and one month after the operation compared to on-pump coronary surgery. According to results from our study, it can be concluded that the early differences in HRV between off-pump versus on-pump CABG patients disappear few months after coronary surgery. Even though off-pump patients had higher preoperative EF than on-pump patients, that did not have significant impact on obtained postoperative values of HRV variables in this study. Also, we did not find any significant differences in postoperative EF and functional capacity in off-pump versus on-pump CABG patients. 

 The limitation of this research is that it was not a randomized study. Nevertheless, the study was performed in the rehabilitation centre in which patients come to in a few weeks or months after CABG, so the randomization was not possible to perform. Also, HRV was not analyzed in patients before and very early after coronary surgery, but, the aim of this study was to analyze autonomic heart function few months after CABG. In spite of that, we believe that the results of this study are worthwhile because we directly compared HRV changes in off-pump versus on-pump CABG patients in a few months after surgery, and just those literature data lacked.

## 5. Conclusions

In conclusion, the results of the study showed that there are no differences in HRV few months after surgery between patients operated on with off-pump versus on-pump CABG.

## Figures and Tables

**Table 1:** Differences in basic characteristics: on-pump versus off-pump CABG patients.

Characteristics of patients	On-pump CABG (*N* = 140)	Off-pump CABG (*N* = 66)	*P*
Age (years)	61 ± 8	60 ± 8	NS
Gender (M/F)	113/27	52/14	NS
BMI (kg/m^2^)	27 ± 2	27.7 ± 3	NS
Former smokers	58(41%)	25(38%)	NS
Smokers	5(4%)	2(3%)	NS
Arterial hypertension	99(71%)	49(74%)	NS
Dyslipidemia	118(84%)	53(80%)	NS
Diabetes mellitus	40(29%)	20(30%)	NS
Previous myocardial infarction	82(59%)	39(59%)	NS
EF before operation	55 ± 9	58 ± 8	*0.01*
Total complications after operation	68(49%)	29(44%)	NS
* *(a) Paroxismal atrial fibrillation	25(18%)	10(15%)	NS
* * (b) Infection	24(17%)	7(11%)	NS
* * (c) Acute renal insufficiency	8(6%)	3(5%)	NS
* * (d) Pleural effusion	34(24%)	11(17%)	NS
* * (e) Stroke	4(3%)	2(3%)	NS
* * (f) Perioperative myocardial infarction	6(4%)	3(5%)	NS

**Table 2:** Differences in heart rate variability between off-pump versus on-pump CABG patients.

HRV variables	Off-pump CABG Mean ± SD	On-pump CABG Mean ± SD	*P*
Mean RR interval (milliseconds)	885 ± 106	879 ± 125	NS
SDNN (milliseconds)	107 ± 30	105 ± 34	NS
SDNN-i (milliseconds)	39 ± 14	41 ± 19	NS
SDANN-i (milliseconds)	96 ± 27	93 ± 32	NS
rMSSD (milliseconds)	24 ± 12	28 ± 18	NS
pNN50 (%)	4.8 ± 6.4	5.7 ± 8.1	NS
TP (ms^2^)	2298 ± 2472	2156 ± 1913	NS
VLF (ms^2^)	1397 ± 1117	1345 ± 988	NS
LF (ms^2^)	302 ± 341	384 ± 409	NS
HF (ms^2^)	262 ± 292	216 ± 261	NS
LF/HF	2.7 ± 1.9	2.5 ± 1.8	NS

Mean RR—mean of R-R intervals for normal beats, SDNN-standard deviation of all normal R-R intervals, SDANN-i—standard deviation of the 5-minutes means of R-R intervals, SDNNi—mean of the 5-minute standard deviations of RR intervals, rMSSD—square root of the mean of the squared successive differences in R-R intervals and pNN50-percentage of R-R intervals that are at least 50 milliseconds different from the previous interval, TP—Total power (0.0–0.5 Hz), VLF—very low (0.003–0.04 Hz), LF—low (0.04–0.15 Hz) and HF—high (0.15–0.4 Hz) frequency components, LF/HF—low to high frequency ratio.
